# 1378. Impact of Cytomegalovirus Prophylaxis on Clinical Outcomes in Kidney Transplantation: A United States Renal Data System-Medicare Linked Database Study

**DOI:** 10.1093/ofid/ofab466.1570

**Published:** 2021-12-04

**Authors:** Amit D Raval, Michael Ganz, Priya Saravanan, Yuexin Tang, Carlos Santos

**Affiliations:** 1 Merck and Co., Inc., Rahway, New Jersey; 2 Evidera, Inc., Waltham, Massachusetts; 3 Merck and Co., Inc, North wales, Pennsylvania; 4 Rush University Medical Center, Chicago, Illinois

## Abstract

**Background:**

Guidelines recommends cytomegalovirus (CMV) prophylaxis by CMV serostatus/risk status, as the currently available antiviral agents may lead to myelosuppressive events in kidney transplant recipients (KTRs). Limited data exist for the United States (US) on the such clinical outcomes with CMV prophylaxis KTRs especially stratified by CMV risk strata. We examined the associations between clinical outcomes and CMV prophylaxis among adult KTRs stratified by CMV risk strata.

**Methods:**

We employed a retrospective cohort design using the US Renal Data System registry-linked Medicare data (2011-2017). The cohort included 22,918 adult KTRs with continuous Medicare Part A & B coverage for ≥ 6-month pre and ≥ 12-month post KT and Part D coverage for ≥ 12-month post- KT. CMV prophylaxis was defined as ≥ 1 prescription fill or medical claim for valacyclovir or valganciclovir at prophylaxis doses within 28 days post-KT.

**Results:**

CMV prophylaxis was utilized by 75% of the cohort. In no CMV prophylaxis group, 52.2% and 34.2% of high and intermediate risk KTRs received valganciclovir (as either pre-emptive or deferred therapy), respectively. Among high risk KTRs, CMV prophylaxis group had significantly lower proportions of KTRs with CMV infection, opportunistic infections (OIs) including bacterial, and fungal infections, and new onset of diabetes mellitus (NODAT) compared to no prophylaxis group. There were no differences in the rates of acute rejection or death; however, a trend towards lower rate of graft-failure at 12-month post-KT. Nearly 40% of high-risk KTRs had myelosuppressive events (leukopenia: 18%; neutropenia:15% thrombocytopenia :19%); however, their differences were non-significant except for thrombocytopenia by CMV prophylaxis status (**Table 1**). CMV infection and myelosuppressive event rates were higher in high-risk than intermediate/low risk KTRs irrespective of CMV prophylaxis status.

**Conclusion:**

CMV prophylaxis was associated with lower rates of CMV infection, OIs, NODAT and graft failure compared to no prophylaxis, however, the burden of CMV infection, OIs and myelosuppression was greater in high-risk KTRs indicating further research is needed on factors associated with greater disease burden in high-risk KTRs.

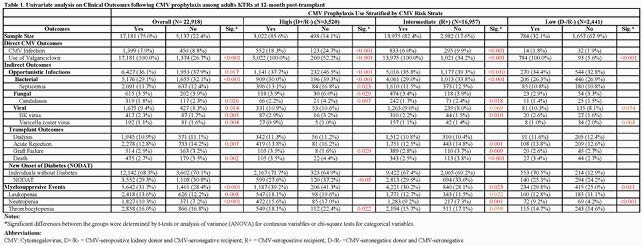

Table 1

**Disclosures:**

**Amit D. Raval, PhD**, **Merck and Co., Inc.** (Employee) **Yuexin Tang, PhD**, **JnJ** (Other Financial or Material Support, Spouse’s employment)**Merck & Co., Inc.** (Employee, Shareholder)

